# Supplementary material for: Novel Driver Strength Index highlights important cancer genes in TCGA PanCanAtlas patients
Source: PeerJ. 2022 Aug 11;10:e13860. doi: 10.7717/peerj.13860 (PMC9375969; doi:10.7717/peerj.13860)
Supplement: Supplemental Information 2 [file peerj-10-13860-s002.zip › COHORTS/genes plots/cohorts/2021_8_16_14_9_distribution_cohorts_males_LGG.pdf]

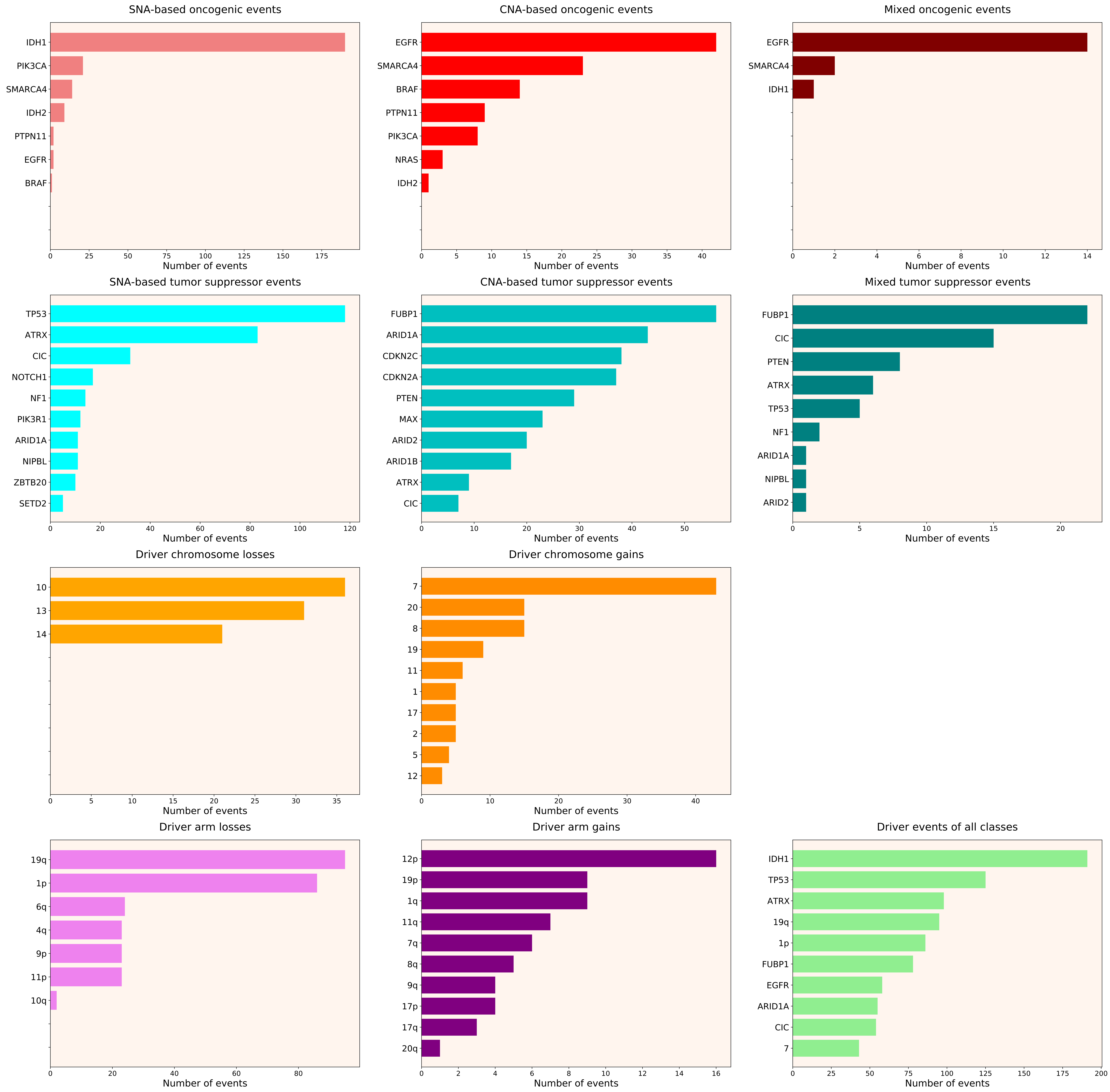

SNA-based oncogenic events
IDH1
PIK3CA
SMARCA4
IDH2
PTPN11
EGFR
BRAF
0 25 50 75 100 125 150 175
Number of events
CNA-based oncogenic events
EGFR
SMARCA4
BRAF
PTPN11
PIK3CA
NRAS
IDH2
0 5 10 15 20 25 30 35 40
Number of events
Mixed oncogenic events
EGFR
SMARCA4
IDH1
0 2 4 6 8 10 12 14
Number of events
SNA-based tumor suppressor events
TP53
ATRX
CIC
NOTCH1
NF1
PIK3R1
ARID1A
NIPBL
ZBTB20
SETD2
0 20 40 60 80 100 120
Number of events
CNA-based tumor suppressor events
FUBP1
ARID1A
CDKN2C
CDKN2A
PTEN
MAX
ARID2
ARID1B
ATRX
CIC
0 10 20 30 40 50
Number of events
Mixed tumor suppressor events
FUBP1
CIC
PTEN
ATRX
TP53
NF1
ARID1A
NIPBL
ARID2
0 5 10 15 20
Number of events
Driver chromosome losses
10
13
14
0 5 10 15 20 25 30 35
Number of events
Driver chromosome gains
7
20
8
19
11
1
17
2
5
12
0 10 20 30 40
Number of events
Driver arm losses
19q
1p
6q
4q
9p
11p
10q
0 20 40 60 80
Number of events
Driver arm gains
12p
19p
1q
11q
7q
8q
9q
17p
17q
20q
0 2 4 6 8 10 12 14 16
Number of events
Driver events of all classes
IDH1
TP53
ATRX
19q
1p
FUBP1
EGFR
ARID1A
CIC
7
0 25 50 75 100 125 150 175 200
Number of events
